# The modifiable areal unit problem (MAUP) in the relationship between exposure to NO_2 _and respiratory health

**DOI:** 10.1186/1476-072X-10-58

**Published:** 2011-10-31

**Authors:** Marie-Pierre Parenteau, Michael C Sawada

**Affiliations:** 1 Laboratory for Applied Geomatics and GIS Science (LAGGISS), Department of Geography, University of Ottawa, Simard Hall, 60 University, Ottawa, K1N 6N5, Canada

## Abstract

**Background:**

Many Canadian population health studies, including those focusing on the relationship between exposure to air pollution and health, have operationalized neighbourhoods at the census tract scale. At the same time, the conceptualization of place at the local scale is one of the weakest theoretical aspects in health geography. The modifiable areal unit problem (MAUP) raises issues when census tracts are used as neighbourhood proxies, and no other alternate spatial structure is used for sensitivity analysis. In the literature, conclusions on the relationship between NO_2 _and health outcomes are divided, and this situation may in part be due to the selection of an inappropriate spatial structure for analysis. Here, we undertake an analysis of NO_2 _and respiratory health in Ottawa, Canada using three different spatial structures in order to elucidate the effects that the spatial unit of analysis can have on analytical results.

**Results:**

Using three different spatial structures to examine and quantify the relationship between NO_2 _and respiratory morbidity, we offer three main conclusions: 1) exploratory spatial analytical methods can serve as an indication of the potential effect of the MAUP; 2) OLS regression results differ significantly using different spatial representations, and this could be a contributing factor to the lack of consensus in studies that focus on the relation between NO_2 _and respiratory health at the area-level; and 3) the use of three spatial representations confirms no measured effect of NO_2 _exposure on respiratory health in Ottawa.

**Conclusions:**

Area units used in population health studies should be delineated so as to represent the *a priori *scale of the expected scale interaction between neighbourhood processes and health. A thorough understanding of the role of the MAUP in the study of the relationship between NO_2 _and respiratory health is necessary for research into disease pathways based on statistical models, and for decision-makers to assess the scale at which interventions will have maximum benefit. In general, more research on the role of spatial representation in health studies is needed.

## Background

The neighbourhood concept is equivocal. Neighbourhood units are often defined as small-areas that share some predefined set of characteristics [1,2]. Neighbourhood definition is an issue in many health studies at the intra-urban level that depend on this geographical concept. In Canada, the neighbourhood has been operationalized as the census tract in several studies [3-9], even if the use of this geographic unit is questionable. Only a few Canadian studies have specifically operationalized the neighbourhood in the context of health research [10-12]. As a consequence, exactly how place is conceptualized at the local scale is one of the weakest theoretical aspects of the way health studies, among others, are currently conducted [13]. The study of the relationship between exposure to criteria pollutants, such as nitrogen dioxide (NO_2_) and health outcomes is no exception to this problem. Unfortunately, few health studies have focused on this issue [14], despite existing literature that demonstrates the feasibility of measuring different levels of association between health and space under different spatial zoning systems [15-18]. Considering that studies on the relationship between NO_2 _and health outcomes are divided regarding the role of exposure to NO_2 _on health, it is surprising that the study of the role of spatial representation in the analysis of this relationship has not received more attention.

Standard geographical units from the Canadian Census, especially the census tract, are often used to operationalize the neighbourhood concept. This method finds benefit in the readily available Census data for this zoning system [19]. In Canada, census tracts are delineated based on optimal population counts, the compactness of the shape, visible boundaries and input from local experts [20]. The census tract boundaries must follow visible features when possible, but in some cases, they are delineated by administrative boundaries and as such census tract definitions can be equivocal [21,22]. Homogeneity in terms of socioeconomic status is not part of the boundary delineation criteria for census tracts or other standard geographical classification levels used in 2006 Canadian Census geography [21,23]. However, neighbourhood units may be expected to be homogeneous along those socioeconomic dimensions related to a given health outcome(s) [1]. Consequently, the use of a census tract as a neighbourhood proxy becomes conceptually problematic. From an analytical viewpoint, using census tracts as the only spatial unit of measure is questionable when no other alternative spatial structure is used for sensitivity analysis, in which case, there can be no assessment of MAUP effects on results [16,21].

The Modifiable Area Unit Problem (MAUP) can cause differences in the analytical results of the same input data compiled under different zoning systems [16,24,25]. Openshaw [16] explains that the MAUP is composed of a scale effect and a zoning effect. Herein, we use the term spatial structure to designate a particular combination of scale and zoning within a bounded region. The scale effect arises when the size of the spatial units of measure changes due to spatial aggregation procedures. Differing spatial aggregation schemes affect analytical results on the same dataset. The zoning effect arises when the number of the spatial units of measure remains the same, but changing their relative structure (changes in the unit boundaries and shape) generates different analytical results [14,26]. According to some authors [23,27], any study about the association between health and place will be influenced by the scale and zoning design used to conduct the study. Generally, the scale effect is recognized as the most troublesome component of the MAUP, while the zoning effect (effect of unit shape) matters to a lesser extent [23,25]. However, the interplay between zoning and scale is complex because either effect can vary in weight due to the spatial scale of the process(es) being analyzed.

The MAUP impacts the results of univariate and multivariate regressions [25]. The MAUP can negatively affect regression model calibration and lead to unreliable results [26]. Some authors have provided insights as to the cause of the MAUP in regression analysis [28]. Accordingly, the MAUP "may be caused by the spatial non-stationarity of multiple predictors that together may be factors for a response variable" [29]. Because the health status of an individual is the result of multiple factors that vary at different spatial scales across a geographic region, health studies are at increased risk of being affected by the MAUP [29,30]. One way to mitigate the impact of the MAUP on analytical results is to create a geographical structure with zoning units that possess "high internal homogeneity" and maintain a considerable amount of external or between-unit heterogeneity [31]. Such mitigation can also represent a solution to errors in the model building process that are induced by positive spatial autocorrelation [32]. Another proposed solution requires the evaluation of the association under different spatial structures (varying zoning and scale) as a way to conduct sensitivity analysis [26].

An automated zone design methodology was first developed for the study of zoning and scale effects on various analytical results [15]. These automated approaches, based on computer algorithms, regroup a set of spatial units into a number of zones so that each unique spatial unit is linked to one zone only [15,33]. A contiguity/adjacency constraint is often used in these models [15]. Examples of other constraints include the compactness (shape), population count, area size and internal homogeneity. Automated zoning has been used for the study of the relationship between morbidity and deprivation [27]. Results of such experiments indicate that automatically delineated zoning systems that increase spatial aggregation tend to produce stronger correlations over smaller census zones [27]. Observations of increasing strength of statistical relationships with increasing spatial aggregation verify the work of Openshaw [15].

Since the main objective of this research is to determine the impact of the MAUP on the study of the relationship between exposure to NO_2 _and respiratory health, three different spatial structures are incorporated into our framework: First, census tracts from the 2006 Canadian Census of population are used as small-scale basic administrative units; second, coarser natural neighbourhoods are delineated based on a homogeneity criteria in order to represent an optimal zoning design for the socioeconomic processes under consideration, and; third, an automated zoning structure is created through a continuity based aggregation of census tracts in order to present a different zoning structure with a scale equivalent to the natural neighbourhoods. By comparing analytical results from three spatial structures, we will improve our understanding of how scale and zoning influence the measured relationship between NO_2 _and health.

## Methods

### Health Outcome Data

The effect of air pollution on respiratory health can be measured through emergency room visits and hospital admissions [34]. The respiratory morbidity rate for individuals 15 years of age and over from the Ottawa Public Health Unit is the primary health outcome measure in this research.

Health conditions associated with exposure to NO_2 _were identified from the International Classification of Disease 10^th ^Revision (ICD-10) [35] based on literature regarding the health effects of air pollution [36]. All records with a principal diagnosis of chronic lower respiratory disease (codes J40-J47) were selected from the *Discharge Abstract Database *(DAD) and the *National Ambulatory Care Reporting System *(NACRS) for fiscal years 2005-2006, 2006-2007 and 2007-2008 and compiled at the geographic level of the census tract and 95 Ottawa neighbourhoods. The Ontario DAD dataset contains "demographic, administrative and clinical data for hospital discharges" and day procedures [37]. NACRS contains "demographic, administrative and clinical data for ambulatory care visits" [38]. Morbidity rates were directly sex and age standardized (45, 48) for age groups 15-24, 25-34, 35-44, 45-54, 55-64, 65-74, 75-84 and 85 and over.

**Figure 1:**
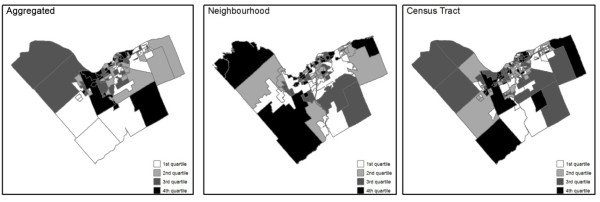
**Spatial distribution of the morbidity rates**. *Respiratory health outcome rate *for each spatial structure (using the quartile for classification).

The spatial distributions of the *Respiratory health outcome rate *for each of the spatial structures are shown in Figure 1.

### Natural neighbourhood, aggregated structure and census tract

The natural neighbourhoods were delineated through a semi-automatic approach with the purpose of being used as the geography of reference for this research as well as for the Ottawa Neighbourhood Study (ONS) project [39]. The objective was to delineate homogeneous units in terms of socioeconomic status (SES), which has been linked to health outcomes, which would also maximize external heterogeneity. The socioeconomic variables from the 2001 Canadian Census of population that were used in the neighbourhood boundary delineation included:

• *Median household income*

• *Unemployment rate*

• *Housing affordability*

• *% Structures built before 1961*

• *% Dwelling owned*

• *Median value of dwelling*

• *% Visible minority*

• *% Population with a bachelor's degree*

The first step involved automatically aggregating dissemination areas (DAs) through spatially constrained clustering and wombling using the software BoundarySeer [40]. The clustering and wombling algorithms were applied to SES and housing data from the 2001 Canadian Census at the geographic level of the dissemination area (DA), "the smallest standard geographic area for which all census data are disseminated" [41]. Once the automated delineation was completed, the natural neighbourhoods were manually refined. To better represent Ottawa's neighbourhoods, some boundaries were later updated following the release of 2006 Census data. A total of 95 neighbourhood units are within the boundary file. This iterative boundary delineation work was achieved through consultations with the City of Ottawa and leaders of local grassroots organizations and public input. Details on the methodological approach are published elsewhere [39].

The aggregated structure was created through the grouping of census tracts using ArcGIS 9.2 [42] with a simple contiguity constraint. This aggregated set was constructed by first selecting a census tract at random and then unioning it with a neighbour before moving to the following census tract that was not already part of an aggregated set. This process was repeated until all non-previously aggregated census tracts were visited. We automatically delineated 95 units in total to compare with 95 natural neighbourhoods in order to evaluate the zoning effect. The final spatial structure used in this research is from the 2006 Canadian Census. From the census, 184 census tracts were extracted to cover the study area in order to represent the data and measures at a more detailed spatial scale. The use of these three spatial structures allows for the assessment of the scale effect and the zoning effect in the study of the relationship between exposure to NO_2 _and health.

The social, economic and environmental settings where an individual lives are contextual variables in ecological studies that can mediate individual level health [43-46]. The explanatory variables at the geographic level of the census tract, the natural neighbourhood and the aggregated structure were obtained from the 2006 Canadian Census of Population. In the case of the data at the geographic level of the neighbourhood, the data were obtained through a custom tabulation from Statistics Canada.

### Exposure measures

**Figure 2:**
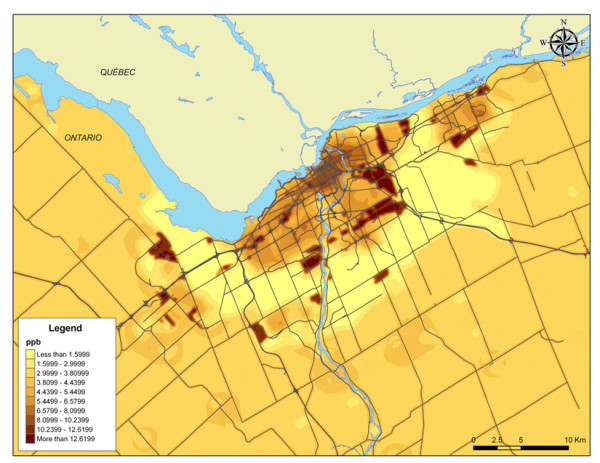
**NO_2 _concentrations (ppb) in the study area**. Results of the LUR modelling for the mapping of NO_2 _concentrations in Ottawa, Canada.

A land-use regression (LUR) model was developed for the mapping of NO_2 _concentrations in Ottawa, Canada (Figure 2). Details of that model are published elsewhere [47]. The model, which included data on the road network, population, green spaces and industrial land-use, yielded an R^2 ^of 0.8055. Zonal statistics within ArcGIS 9.2 were derived from the LUR modelled NO_2 _layer to derive mean NO_2 _concentrations for the census tracts, natural neighbourhoods and aggregated structure [48].

## Results

Preliminary data analysis was first conducted to determine the role of spatial representation on summary statistics using global spatial autocorrelation and bivariate Moran's I. Bivariate Ordinary Least Squares (OLS) and multivariate OLS regressions where then applied to the three zoning systems to determine the impact of the MAUP on the relationship between the *Respiratory health outcome rate *and exposure to NO_2_. Spatial regressions were not used in this research because a comparison between spatial regression and OLS has already been explored in another article in preparation by the same authors. SAS (version 9.2) [49] and GEODA [50] were used for statistical and spatial analysis of the data.

### Summary statistics

The mean value of the explanatory variables under each of the three spatial structures is similar (Table 1). The variables *Educational attainment *and *% low income *have the highest levels of variability. The variable *Mean NO_2 _concentration *displays a much smaller amount of variability under different spatial structures, with average values ranging from 5.29 to 5.39 ppb. On the other hand, the mean value for the *Respiratory health outcome rate *is significantly affected by the use of different spatial structures. The lowest average rate is 1,275.35 per 100,000 at the natural neighbourhood level in comparison to 2,346.89 per 100,000 for the census tract structure. Standard deviations are also similar for most variables under three different spatial structures, but as with the mean values, there are exceptions. Finally, census tracts have higher variance values then the other two spatial structures.

**Table 1:** Summary statistics

	Natural Neighbourhood (n = 95)					Aggregated (n = 95)					Census tract (n = 184)				
Variable	Mean	Std Dev	Min	Max	Variance	Mean	Std Dev	Min	Max	Variance	Mean	Std Dev	Min	Max	Variance
% Occupied private dwellings in need of major repair	5.86	3.29	0.37	13.48	10.83	6.07	3.41	0.96	13.55	11.63	6.06	3.89	0.00	15.73	15.11
% Occupied private dwellings built before 1946	7.65	13.04	0.00	72.87	170.15	9.08	14.38	0.00	68.43	206.72	9.38	16.52	0.00	77.98	272.78
Unemployment rate of the total population 15 years and over	6.12	1.60	2.90	9.90	2.56	6.04	1.99	1.95	11.66	3.96	5.96	2.39	0.00	15.50	5.71
Educational attainment - no certificate, diploma or degree	15.62	4.71	6.48	31.53	22.22	8.06	4.57	1.05	22.19	20.85	7.97	5.37	0.00	27.66	28.84
Median income (2005)	3.31E+04	5.68E+03	2.02E+04	4.57E+04	3.22E+07	3.43E+04	7.22E+03	1.44E+04	4.85E+04	5.21E+07	3.42E+04	9.24E+03	0.00E+00	5.98E+04	8.54E+07
Average income (2005)	4.34E+04	9.76E+03	2.27E+04	8.14E+04	9.53E+07	4.41E+04	9.64E+03	2.00E+04	7.64E+04	9.29E+07	4.40E+04	1.46E+04	0.00E+00	1.34E+05	2.12E+08
% low income	14.94	10.79	1.55	49.91	116.51	9.23	7.57	0.00	40.20	57.35	8.81	8.71	0.00	51.60	75.78
Mean nitrogen dioxide concentration	5.32	1.80	1.05	10.56	3.24	5.30	1.91	1.03	9.52	3.65	5.40	1.93	0.96	10.18	3.74
Respiratory health outcome rate	1.28E+03	7.83E+02	0.00E+00	3.52E+03	6.13E+05	2.35E+03	1.15E+03	5.50E+02	6.21E+03	1.33E+06	2.35E+03	1.56E+03	0.00E+00	1.20E+04	2.44E+06
% Lone-parent families	17.12	7.09	6.34	38.93	50.30	16.54	6.73	5.74	37.45	45.32	16.19	7.71	0.00	48.35	59.45
% Owned dwellings	67.17	24.61	2.44	98.32	605.74	68.78	24.95	5.22	98.02	622.58	68.06	27.30	0.00	99.23	745.48
% Rented dwellings	32.68	24.65	1.45	97.60	607.79	31.20	24.92	2.12	94.51	620.84	30.86	26.56	0.00	95.31	705.26
% Occupied private dwellings of type single house	44.41	26.43	0.19	96.17	698.73	45.30	26.14	0.27	97.35	683.34	45.14	30.14	0.00	100.00	908.71
% Occupied private dwellings of type row house	20.72	16.66	0.00	63.44	277.59	19.76	15.89	0.00	83.08	252.38	18.90	17.76	0.00	83.08	315.43
% Occupied private dwellings of type apartment (building that has 5 or more stories)	18.26	21.17	0.00	84.91	447.97	16.12	18.44	0.00	73.93	339.92	15.73	21.39	0.00	96.73	457.40
% Did not lived at the same address one year ago	14.49	6.16	4.47	36.44	37.92	14.68	6.47	5.49	35.03	41.89	14.54	7.90	0.00	55.65	62.35
% Did not lived at the same address five years ago	42.86	11.27	19.79	78.48	127.07	43.35	12.48	22.31	82.92	155.79	42.69	15.55	0.00	90.54	241.71
% Walk - mode of transportation to work	7.30	8.85	0.00	52.03	78.33	8.39	10.60	1.01	52.58	112.35	8.20	10.84	0.00	53.36	117.44
% Bicycle - mode of transportation to work	2.18	2.01	0.00	9.78	4.05	2.37	2.17	0.00	12.02	4.73	2.40	2.35	0.00	12.90	5.51
Average value of owned dwelling	2.99E+05	6.82E+04	1.98E+05	5.90E+05	4.65E+09	2.98E+05	6.48E+04	1.68E+05	5.53E+05	4.20E+09	2.99E+05	9.23E+04	0.00E+00	9.36E+05	8.52E+09
Participation rate of the total population 15 years and over	68.38	6.45	47.74	80.87	41.66	69.25	6.30	53.52	81.12	39.74	68.17	10.21	0.00	82.70	104.23
% Management occupations	11.37	3.26	4.99	20.00	10.62	11.72	3.23	5.34	21.69	10.43	11.57	4.03	0.00	21.69	16.26
% Retail trade industry	10.23	2.81	2.73	17.36	7.88	10.23	2.62	5.28	19.07	6.86	10.09	3.39	0.00	20.88	11.49

Summary statistics of the explanatory variables and the dependent variable under the three spatial structures under study.

### Global spatial autocorrelation

Global Moran's I spatial autocorrelation statistic measures the self-similarity of a spatial variable's value as a function of adjacency [51]. Using a first-order Queen's case spatial weight matrix and 999 permutations, we found statistically significant spatial autocorrelation at a pseudo-significance level of *p *≤ 0.05 for all the explanatory variables for all spatial structures, with the exception of *Average income *within the natural structure (Table 2). The exposure data (*Mean NO_2 _concentration*) is characterized by strong statistically significant spatial autocorrelation under all spatial structures. The *Respiratory health outcome rate *exhibits significant spatial autocorrelation within the census and aggregated structures but not within natural neighbourhood boundaries.

**Table 2:** Global spatial autocorrelation

Variable	Natural Neighbourhood (n = 95)	Aggregated (n = 95)	Census Tract (n = 184)
% Occupied private dwellings in need of major repair	0.4337 (0.001)	0.5604 (0.001)	0.4800 (0.001)
% Occupied private dwellings built before 1946	0.5196 (0.001)	0.6182 (0.001)	0.5948 (0.001)
Unemployment rate of the total population 15 years and over	0.4695 (0.001)	0.3192 (0.001)	0.2367 (0.001)
Educational attainment - no certificate, diploma or degree	0.2518 (0.002)	0.3468 (0.001)	0.3397 (0.001)
Median income (2005)	0.3932 (0.001)	0.2489 (0.001)	0.2397 (0.001)
Average income (2005)	0.0561 (0.145)	0.1022 (0.045)	0.1139 (0.005)
% low income	0.5134 (0.001)	0.4518 (0.001)	0.2859 (0.001)
Mean nitrogen dioxide concentration	0.5215 (0.001)	0.5463 (0.001)	0.6656 (0.001)
Respiratory health outcome rate	0.0261 (0.288)	0.1775 (0.009)	0.1315 (0.002)
% Lone-parent families	0.3329 (0.001)	0.4183 (0.001)	0.3206 (0.001)
% Owned dwellings	0.5931 (0.001)	0.6714 (0.001)	0.5796 (0.001)
% Rented dwellings	0.5943 (0.001)	0.6715 (0.001)	0.6029 (0.001)
% Occupied private dwellings of type single house	0.4824 (0.001)	0.5444 (0.001)	0.4164 (0.001)
% Occupied private dwellings of type row house	0.1829 (0.007)	0.2871 (0.001)	0.2814 (0.001)
% Occupied private dwellings of type apartment (building that has 5 or more stories)	0.4607 (0.001)	0.5457 (0.001)	0.4177 (0.001)
% Did not lived at the same address one year ago	0.4538 (0.001)	0.4535 (0.001)	0.3808 (0.001)
% Did not lived at the same address five years ago	0.2851 (0.001)	0.3049 (0.001)	0.3151 (0.001)
% Walk - mode of transportation to work	0.8129 (0.001)	0.7489 (0.001)	0.7801 (0.001)
% Bicycle - mode of transportation to work	0.5865 (0.001)	0.6383 (0.001)	0.6757 (0.001)
Average value of owned dwelling	0.1418 (0.002)	0.1440 (0.013)	0.1547 (0.001)
Participation rate of the total population 15 years and over	0.5157 (0.001)	0.5141 (0.001)	0.2063 (0.001)
% Management occupations	0.1138 (0.045)	0.1763 (0.005)	0.2000 (0.001)
% Retail trade industry	0.1989 (0.002)	0.3199 (0.001)	0.2652 (0.001)

Global Moran's I values for the explanatory variables for the three spatial structures under study. *p*-values are in parentheses.

The comparison of Moran's I within the natural neighbourhoods and aggregated structure reveals a zoning effect. In general the aggregated structure exhibits higher magnitudes of Moran's I. For both structures, spatial autocorrelation is statistically significant for all the variables with the exception of the *Respiratory health outcome rate *and the *Average income *(under the neighbourhood structure). The *Respiratory health outcome rate *is characterized by statistically significant positive spatial autocorrelation within the aggregated structure, but not for the natural neighbourhoods.

A scale effect is observed when comparing results using the aggregated structure and census tracts. The aggregated structure, which is based on an aggregation of census tracts, displays stronger spatial autocorrelation in 70% of the variables when compared to the census tract. Whereas, compared to the natural neighbourhoods, global spatial autocorrelation in census tracts is stronger or weaker in half the cases. As such, the use of homogeneous natural neighbourhoods is apparently compensating for the scale effect evident in the aggregated structure that has equivalently sized units.

### Bivariate Moran's I

Bivariate Moran's I is used here in exploratory spatial data analysis (ESDA) to provide information on the strength of the associations between NO_2 _concentrations and the other covariates as well as between the *Respiratory health outcome rate *and all explanatory variables [52]. This approach also provided preliminary information on the likely direction of the effect [53].

The spatial correlation between the *Respiratory health outcome rate *and explanatory variables differs from one structure to the other and appears to be affected by scaling and the zoning effects (Table 3). For most relations, the direction or sign of the correlation is the same for all three structures. The *Average value of owned dwelling *and *% retail trade industry *are two exceptions. The *Average value of owned dwelling *has a non-significant positive spatial correlation with the *Respiratory health outcome rate *under the neighbourhood structure, but a negative and non-statistically significant for the aggregated and census tract structures. The *% retail trade industry *is negatively correlated with the *Respiratory health outcome rate *under the natural neighbourhood structure (statistically significant) and the census tract structure (not statistically significant) but is positive under the aggregated structure (statistically significant).

**Table 3:** Bivariate Moran's I

	Natural Neighbourhood (n = 95)		Aggregated (n = 95)		Census Tract (n = 184)	
Variable	Mean NO_2_	Resp health	Mean NO_2_	Resp health	Mean NO_2_	Resp health
% Occupied private dwellings in need of major repair	0.3971 (0.001)	0.1329 (0.001)	0.4776 (0.001)	0.1798 (0.001)	0.4515 (0.001)	0.0899 (0.001)
% Occupied private dwellings built before 1946	0.3235 (0.001)	0.1045 (0.001)	0.4180 (0.001)	0.0636 (0.001)	0.3939 (0.001)	0.0001 (0.999)
Unemployment rate of the total population 15 years and over	0.3741 (0.001)	0.1799 (0.001)	0.3982 (0.001)	0.1446 (0.001)	0.3266 (0.001)	0.0363 (0.087)
Educational attainment - no certificate, diploma or degree	0.1518 (0.001)	0.0589(0.001)	0.2843 (0.001)	0.1609 (0.001)	0.2420 (0.001)	0.0663 (0.001)
Median income (2005)	-0.4019 (0.001)	-0.1598 (0.001)	-0.3765 (0.001)	-0.1111 (0.001)	-0.3031 (0.001)	-0.0583 (0.001)
Average income (2005)	-0.1739 (0.001)	-0.0598 (0.001)	-0.2272 (0.001)	-0.0435 (0.001)	-0.1613 (0.001)	-0.2200 (0.509)
% low income	0.5027 (0.001)	0.1685 (0.001)	0.4933 (0.001)	0.1215 (0.001)	0.4158 (0.001)	0.0302 (0.512)
Mean nitrogen dioxide concentration	1 ()	0.1905 (0.001)	1 ()	0.0656 (0.001)	1 ()	0.0365 (0.085)
Respiratory health outcome rate	0.0794 (0.001)	1 ()	0.1056 (0.001)	1 ()	0.1517 (0.001)	1 ()
% Lone-parent families	0.3353 (0.001)	0.1486 (0.001)	0.4181 (0.001)	0.1716 (0.001)	0.3481 (0.001)	0.0454 (0.700)
% Owned dwellings	-0.5492 (0.001)	-0.2271 (0.001)	-0.6191 (0.001)	-0.1823 (0.001)	-0.5872 (0.001)	-0.0946 (0.001)
% Rented dwellings	0.5507 (0.001)	0.2278 (0.001)	0.6188 (0.001)	0.1824 (0.001)	0.6008 (0.001)	0.0877 (0.001)
% Occupied private dwellings of type single house	-0.4173 (0.001)	-0.2118 (0.001)	-0.4982 (0.001)	-0.2071 (0.001)	-0.4592 (0.001)	-0.0779 (0.001)
% Occupied private dwellings of type row house	-0.2593 (0.001)	-0.0785 (0.001)	-0.2433 (0.001)	-0.0119 (0.999)	-0.2354 (0.001)	-0.0244 (0.087)
% Occupied private dwellings of type apartment (building that has 5 or more stories)	0.4756 (0.001)	0.2116 (0.001)	0.5433 (0.001)	0.1441 (0.001)	0.4728 (0.001)	0.0469 (0.110)
% Did not lived at the same address one year ago	0.4212 (0.001)	0.1737 (0.001)	0.4830 (0.001)	0.1223 (0.001)	0.4114 (0.001)	0.0599 (0.001)
% Did not lived at the same address five years ago	0.2344 (0.001)	0.1044 (0.001)	0.2876 (0.001)	0.0889 (0.001)	0.2155 (0.001)	0.0257 (0.484)
% Walk - mode of transportation to work	0.4795 (0.001)	0.1590 (0.001)	0.5175 (0.001)	0.0858 (0.001)	0.5531 (0.001)	0.0390 (0.117)
% Bicycle - mode of transportation to work	0.4458 (0.001)	0.1576 (0.001)	0.4949 (0.001)	0.1683 (0.001)	0.4553 (0.001)	0.0263 (0.001)
Average value of owned dwelling	0.1167 (0.001)	0.0292 (0.606)	0.0641 (0.001)	-0.0287 (0.098)	0.0509 (0.001)	-0.0360 (0.096)
Participation rate of the total population 15 years and over	-0.3280 (0.001)	-0.1754 (0.001)	-0.3140 (0.001)	-0.1791 (0.001)	-0.1788 (0.001)	-0.1788 (0.001)
% Management occupations	-0.1845 (0.001)	-0.0967 (0.001)	-0.2724 (0.001)	-0.1577 (0.001)	-0.2309 (0.001)	-0.0597 (0.001)
% Retail trade industry	-0.2428 (0.001)	-0.0993 (0.001)	-0.1929 (0.001)	0.0377 (0.0440)	-0.1629 (0.001)	-0.0084 (0.999)

Bivariate Moran's I values 1) between the explanatory variables and the *Mean NO_2 _concentration*; and 2) between the explanatory variables and the *Respiratory health outcome rate*. *p*-values are in parentheses.

The strength of correlation between *Mean NO_2 _concentration *and the *Respiratory health outcome rate *varies according to the spatial structure. For the natural neighbourhoods, bivariate Moran's I is 0.1905 and is statistically significant. The bivariate Morans' I value for the same relationship using the aggregated structure is 0.0656 and is statistically significant. Finally, using census tracts, bivariate Moran's I value is lowest (I = 0.0365) and not statistically significant.

### Bivariate regression

To explore how the scale and zoning affects ordinary least squares, an (OLS) bivariate regression model was developed in GeoDa to measure the relationship between the variable *Mean NO_2 _concentration *and the *Respiratory health outcome rate *for each of the three spatial structures (Table 4). The measured R^2 ^is low for each of the spatial structures: 0.0492 for the census tract structure, 0.0494 for the aggregated structure and 0.0307 with the neighbourhood structure. The value of the *Mean NO_2 _concentration *coefficient is positive for all three spatial structures; it is statistically significant in the aggregated and the census tract models but not in the natural neighbourhood model.

**Table 4:** Bivariate regression

	Natural Neighbourhood (n = 95)	Aggregated (n = 95)	Census Tract (n = 184)
	Bivariate RESP	Bivariate RESP	Bivariate RESP
**R-squared**	0.0307	0.0494	0.0492
**Adjusted R-squared**	0.0202	0.0392	0.0440
**Sum squared residual**	55810400.0000	118990000.0000	424291000.0000
**Sigma-square**	600112.0000	1279470.0000	2330000.0000
**S.E. of regression**	774.6690	1131.1400	1526.8500
**Sigma-square ML**	587478.0000	1252530.0000	2305000.0000
**S.E of regression ML**	766.4710	1119.1700	1518.5300
**F-statistic**	2.9462	4.8363	9.4154
**Prob(F-statistic)**	0.0894	0.0303	0.0025
**Log likelihood**	-765.7700	-801.7310	-1608.9800
**Akaike**	1535.5400	1607.4600	3221.9500
**Schwarz criterion**	1540.6500	1612.5700	3228.3800
**Intercept coefficient**	869.8488 (0.0007)	1634.0250 (< 0.0001)	1379.7439 (< 0.0001)
**Mean NO_2 _coefficient**	76.2276 (0.0894)	134.3078 (0.0303)	179.136 (0.0024)
**Jarque-Bera**	6.3023 (0.0428)	26.1702 (< 0.0001)	923.152 (< 0.0001)
**Breusch-Pagan Test**	5.1764 (0.0228)	1.5039 (0.2200)	0.1623 (0.6870)
**Koenker-Bassett test**	4.1263 (0.0422)	1.0065 (0.3157)	0.0270 (0.8693)
**Moran's I (error)**	-0.0039 (0.8723)	0.2454 (< 0.0001)	0.1080 (0.0096)
**Lagrange Multiplier (lag)**	0.0003 (0.9842)	12.2297 (0.0004)	5.4663 (0.0193)
**Robust LM (lag)**	0.0112 (0.9155)	0.1264 (0.7221)	0.0668 (0.7959)
**Lagrange Multiplier (error)**	0.0025 (0.9599)	1.0239 (0.3115)	5.4253 (0.0198)
**Robust LM (error)**	0.0133 (0.9079)	13.2537 (0.0013)	0.0257 (0.8724)

Results of the bivariate regressions (OLS) between the *Mean NO_2 _concentration *(mean NO_2_) and the *Respiratory health outcome rate *(RESP) for the three spatial structures under study. *p*-values are in parentheses.

The OLS regression model of the natural neighbourhood structure is characterized by a non-normal distribution of the error term (Jarque-Bera) and non-stationarity between the explanatory variables and the *Respiratory health outcome rate *(Breusch-Pagan and Koenker-Bassett tests). The OLS models for the aggregated and census tract structures are also characterized by a non-normal distribution of the error term (Jarque-Bera) but pass the tests for stationarity (Breusch-Pagan and Koenker-Bassett). Finally, the census tract and aggregated structures are characterized by statistically significant spatial autocorrelation in the residuals using the modified Moran's I test for regression residuals. Additionally, the R^2 ^and adjusted R^2 ^values are smaller for the neighbourhood model than the census tract but according to the log likelihood, Akaike information criterion and the Schwarz criterion the neighbourhood model is a better fit.

### Multivariate regression

Explanatory variables were introduced into a stepwise regression for each of the three spatial structures using SAS 9.2 to determine the best fitting model for each of the zoning systems (Table 5). Since the objective of this research was to study the effect of the MAUP in the relationship between exposure to NO_2 _and health, the variable *Mean NO_2 _concentration *was included in each of the models even despite its lack of statistical significant. All other included variables were statistically significant.

**Table 5:** Multivariate regression

	Natural Neighbourhood (n = 95)	Aggregated (n = 95)	Census Tract (n = 184)
	Multivariate OLS	Multivariate OLS	Multivariate OLS
**R-squared**	0.4306	0.3947	0.4341
**Adjusted R-squared**	0.3848	0.3678	0.4150
**Sum squared residual**	32783700.0000	576190000000.0000	252486000.0000
**Sigma-square**	376824.0000	841798.0000	1426470.0000
**S.E. of regression**	613.8600	917.4960	1194.3500
**Sigma-square ML**	345091.0000	797493.0000	1372210.0000
**S.E of regression ML**	587.4450	893.0250	1171.4100
**F-statistic**	9.3999	14.6759	22.6379
**Prob(F-statistic)**	0.0000	0.0000	0.0000
**Log likelihood**	-740.4980	-780.2880	-1561.2200
**Akaike**	1497.0000	1570.5800	3136.4400
**Schwarz criterion**	1517.4300	1583.3400	3158.9500
**Variables**	n.a.	n.a.	n.a.
	Intercept 5327.4610 (0.0001)	Intercept 738.3682 (0.0192)	Intercept -1013.495 (0.0905)
	Mean NO_2 _concentration -18.0183 (0.6855)	Mean NO_2 _concentration 36.4373 (0.5349)	Mean NO_2 _concentration 30.9425 (0.5706)
	Educational Attainment 78.4534 (0.0002)	% Occupied private dwellings in need of major repairs 179.7687 (0.0007)	% Occupied private dwellings in need of major repairs 177.3213 (< 0.0001)
	Participation Rate -53.0824 (0.0003)	% Occupied private dwellings built before 1946 -29.6505 (0.0118)	% Occupied private dwellings built before 1946 -20.91902 (0.0163)
	% Management occupations -84.1282 (0.004)	Educational Attainment 73.4928 (0.0111)	Educational Attainment 113.1868 (< 0.0001)
	% Walk - mode of transportation to work 40.1355 (< 0.0001)		Average income 0.0151 (0.0442)
	% Occupied private dwellings of type apartment -16.7916 (0.0015)		% Retail trade industry 73.8654 (0.0161)
	% Lone-parent families -34.7820 (0.0157)		
**Jarque-Bera**	0.9239 (0.6300)	44.1697 (< 0.0001)	1173.241 (< 0.0001)
**Breusch-Pagan Test**	3.0898 (0.8765)	8.5867 (0.0723)	149.1566 (< 0.0001)
**Koenker-Bassett test**	3.1269 (0.8730)	3.8557 (0.4258)	21.8956 (0.0012)
**Moran's I (error)**	0.0498 (0.2527)	0.1892 (0.0006)	0.1091 (0.0051)
**Lagrange Multiplier (lag)**	0.1953 (0.6584)	7.8384 (0.0051)	2.7432 (0.0976)
**Robust LM (lag)**	3.4593 (0.0628)	0.6030 (0.4374)	0.2288 (0.6324)
**Lagrange Multiplier (error)**	0.4047 (0.0554)	7.8074 (0.0052)	5.5355 (0.0186)
**Robust LM (error)**	3.6687 (0.0628)	0.5720 (0.4494)	3.0211 (0.0821)

Results of the multivariate regressions (OLS) for the three spatial structures under study. *p*-values are in parentheses.

The model developed for the census tract structure contains six explanatory variables: *% occupied private dwellings in need of major repairs, % occupied private dwellings built before 1946, Educational attainment, Average income, % retail trade industry *and *Mean NO_2 _concentration *and yielded an R^2 ^value of 0.43 and an adjusted R^2 ^of 0.41. The model for the aggregated structure is a subset of the census tract model; it contains four of the variables found in the census tract model and explains less of the variability in the *Respiratory health outcome rate *(R^2 ^= 0.39 and adjusted R^2 ^= 0.37). The model building exercise provided a different set of explanatory variables using the neighbourhood structure. The model is made up of seven variables (*Educational attainment, Participation rate of the total population 15 years and over, % management occupations, % walk - mode of transportation to work, % occupied private dwellings of type apartment, % lone-parent families*, and *Mean NO_2 _concentration*). The only common variable to the three models, with the exception of *Mean NO_2 _concentration*, is *Educational attainment*. The R^2 ^(0.43) and the adjusted R^2 ^(0.38) for the neighbourhood structure lay between the values generated by the OLS models for the census tract and the aggregated structure.

The log likelihood is larger for the natural neighbourhood than for the census tract and aggregated structures while the AIC and Schwarz criterion have smaller values for the natural neighbourhood structure than the two others, all characterizing the neighbourhood model as an improved fit. The model developed under the natural neighbourhood structure is the only one not characterized by a non-normal distribution of the error term (Jarque-Bera) and non-stationarity between the explanatory variables and the dependent variables (Breusch-Pagan and Koenker-Bassett tests). In terms of global spatial autocorrelation, only the natural neighbourhood model is not characterized by statistically significant spatial autocorrelation.

## Discussion

Numerous studies have concluded that increased exposure to NO_2 _likely contributes to negative respiratory health but no positive and statistically significant association has been unequivocally found [43,54,55]. The main objective of this research was to examine the role of spatial representation, focusing on MAUP effects, in the study of the relationship between exposure to NO_2 _and respiratory health. We find that the outcomes of this exposure/health relation are tied to the MAUP. The MAUP provides a method to learn how scale and zoning may affect the lack of consensus in studies that aim to expose the role that NO_2 _has on overall health outcomes.

Some have recommended approaches to gain a better insight into how the MAUP can affect analytical results [16,56,57] but few published studies have incorporated any of these in their analyses. In our research, we implemented two of the recommended approaches [26]. The first approach is based on the use of an optimal zoning proposal, while the second suggests conducting sensitivity analysis using alternate spatial structures. In this study, the natural neighbourhoods delineated through a semi-automated method were used as the "optimal" zoning system. As mentioned, the main component of the delineation process was based on the concept of internal homogeneity along socioeconomic dimensions. Neighbourhoods were delineated in order to make homogeneous units in terms of SES. These units may not be suitable for all studies conducted in Ottawa, but they are assumed to represent many social processes associated with health. We also believe that these natural neighbourhoods could be used for research examining other health outcomes, aside from respiratory morbidity or even other social processes related to SES.

This research also used sensitivity analysis to mitigate the effect of the MAUP [16,56,57]. This study was conducted using three spatial structures: natural neighbourhoods, an aggregated structure of the same scale as the former but with a different zoning and census tracts with a different scale from the former structures. Using this sensitivity analysis method allowed us to address a number of questions related to the effect that the MAUP has on spatial autocorrelation and hence on univariate and bivariate and regressions.

Exploratory and confirmatory data analysis methods were used to assess the role of spatial representation in the study of the relationship between exposure to NO_2 _and the respiratory health outcomes. The results obtained from the different analytical approaches converge towards three main conclusions, which will be discussed in more detail:

1. Exploratory analytical methods, such as univariate Moran's I and bivariate Moran's I, can serve as an indication of the potential effect of the MAUP in the study of the relationship between exposure to NO_2 _and health;

2. Bivariate and multivariate regressions suggest that different spatial representations can contribute to the lack of consistency of previous literature regarding the relationship between exposure to NO_2 _and health;

3. Results from all three different spatial representations confirm no significant effect of NO_2 _exposure on respiratory health in Ottawa that is not due to unreasonable spatial units of measurement.

The results obtained confirm the documented effect of the MAUP [58,59] on summary statistics. The analysis of the summary statistics demonstrates that the MAUP does not have a strong effect on the mean of explanatory variables, confirming the previous results of other researchers [26]. Our results further substantiate the work of those authors who observed that the variance decreases with increasing aggregation. Likewise, we observe that the variance is affected when the same number of spatial units of study are used but with a different partitioning or zoning of space.

Global Moran's I was calculated for all the explanatory variables as well as for the dependent variable within three spatial structures. All the explanatory variables were characterized by positive spatial autocorrelation within the three zoning systems. Moran's I values tend to be lower for census tracts than the other two spatial structures. As aggregation increases, Moran's I is also expected to increase due to the "increased homogeneity in the landscape structure" [26]. While not definitive, our results concur.

The dependent variable *Respiratory health outcome rate *displays low levels of statistically significant spatial autocorrelation using census tracts and the aggregated structure. Using natural neighbourhoods, global Moran's I calculated for the dependent variable, reveals a non-significant value close to zero. In this case, instead of spatial autocorrelation increasing with aggregation, it is no longer statistically significant. By way of explanation, the natural neighbourhoods are internally homogeneous in terms of SES and adequately depict the spatial scale of the *Respiratory health outcome rate*, thus reducing spatial dependence between neighbourhoods. A zoning effect is also observed; levels of spatial autocorrelation using the natural neighbourhood structure are reduced when compared to the aggregated structure. These observations serve as a rationale for using custom geography delineated using processes known *a priori *to be associated with the dependent health outcome variable of interest.

Under the MAUP, we expected both the aggregated and neighbourhood structures (n = 95) to be characterized by stronger bivariate Moran's I values between the explanatory variables and the *Mean NO_2 _concentration *then under the census tract structure (n = 184) because aggregation is known to increase the strength of correlation [16,27]. The use of an optimal zone design in the natural neighbourhoods based on minimizing internal homogeneity appears to be reducing the scale effect of the MAUP. For half of the explanatory variables, the correlation with *Mean NO_2 _concentration *is stronger at the census tract level than at the neighbourhood level or *vice versa*. On the other hand, the correlations measured using the aggregated structure are stronger (70% of the variables have stronger correlation) than under the census tract structure, which is the expected result [16]. Moreover, a relatively strong zoning effect is observed when comparing the bivariate Moran's I values for the correlation between NO_2 _and health for the neighbourhood and aggregated structures. Similar results were obtained for the correlations between the explanatory variables and the *Respiratory health outcome rate*. This confirms that the natural neighbourhoods, which are internally homogeneous from a socioeconomic perspective, better capture the processes linked to respiratory health outcomes. We also found that the variable *% retail trade industry *has changed directions depending on the spatial structure, confirming that "correlation inference is not robust to the aggregation process" [60].

The use of an exploratory analytical approach in the context of sensitivity analysis can be seen as a tool to assess the role of the MAUP prior to conducting confirmatory data analysis. Exploratory analysis allowed us to obtain a more in-depth understanding of the potential effect of the MAUP on the results of statistical modeling used in the study of the relationships between area-level health and other variables [61].

The results of bivariate and multivariate regression suggest that using different spatial representations could contribute to the lack of consensus found in the literature regarding the NO_2 _and health relation at the area-level. Bivariate regressions between the NO_2 _concentrations and the dependent variable once again confirmed both the zoning effect and the scale effect of the MAUP. According to the census tract and aggregated structures, there is a low but statistically significant relationship between *Mean NO_2 _concentration *and *Respiratory health outcome rate*. As expected from earlier work on the MAUP, the R^2 ^value is higher when using an aggregated structure (coarser level of aggregation) then with the census tract [29]. The relationship measured between NO_2 _and *Respiratory health outcome rate *cannot be confirmed using the neighbourhood structure in this study as it is not statistically significant. The OLS model for the neighbourhood structure is the only model not characterized by statistically significant positive spatial autocorrelation in the residuals and passes all tests for non-normality of the error term and heteroskedasticity, suggesting this model is correctly fitted.

The effect of the MAUP on multivariate analyses has been described as "complex and unpredictable" [26]. Considering that the health of an individual is the result of various factors apart from exposure to NO_2 _[62], the relationship is difficult to measure. The question of which variables are significant within our multivariate regression models is an essential element to be addressed; there was only one variable common to all three models (aside from *Mean NO_2 _concentration *which was forced into the models). The variable *Educational attainment *is an explanatory variable for variations in the morbidity rate for all three structures. In other words, scale and/or the zoning design considerations that may mediate this variable's inclusion/exclusion are being filtered out. The geographic scale of the variable *Educational attainment *is probably larger than the scale of the census tract. Another comparison to be made is between the variables used as input into the delineation of the optimal zoning design and the variables included in the different models. We observe that the census tract model includes both *% occupied private dwellings built before 1946 *and *Average income*, which were used in the delineation of the natural neighbourhoods. By creating an optimal zoning system based on these variables and other SES variables, housing and income covariates are no longer confounding variables in the relationship between exposure to NO_2 _and health and hence are not found in the neighbourhood model.

The variable *Mean NO_2 _concentration *is not a statistically significant factor in any model and so allowed other variables to express their association with respiratory health. For example, *% Lone-parent families*, a measure of the economic structure, is part of the optimal zoning design model based on the natural neighbourhoods but not the census tract or aggregated structures. If the process of aggregation has the same impact on dependent and independent variables, then the effect of the MAUP is reduced in severity [25]. These results are demonstrated when the independent and dependent variables are spatially autocorrelated and averaged. Since the level of global spatial autocorrelation was found to be different for the dependent variable as a function of the spatial structure, the differences observed between the models are justified. Among the variables included in the neighbourhood model, two variables have a coefficient with a direction opposite to that suggested by bivariate Moran's I (*Mean NO_2 _concentration *and *% Lone-parent families*). Finally, the resemblance between the census tract and the aggregated structure; the aggregated structure is a complete subset of the census tract model. Aggregation can potentially generate collinearity between independent variables [29].

The MAUP implies that an OLS model developed using a specific spatial structure should not be transferred to another spatial structure with the same expectations. This finding has implications for research that may use index mapping techniques to estimate community vulnerability to air pollutants. Moreover, this finding has significant implications for studies that aim to propose morbidity pathways using variables that are found to be significant in models tested at only one scale. Thus, there is the prospect that different scales of analysis will deliver markedly different sets of explanatory variables induced by the MAUP. The sensitivity analysis conducted in this research clearly demonstrates that the explanatory factors of respiratory health will vary according to the geographical structure used to conduct the study. Since research relating exposure to NO_2 _and health uses a variety of geographical units to conduct the analysis, the variability of the results of previous research may be caused by the MAUP. We believe the delineation of a custom geography that coincides with the spatial scale of the phenomenon under investigation can be justified because there is no prior reason to believe that administrative and statistical boundaries reflect the fundamental nature and scale of the economic and social phenomena measured within them [25]. The use of optimal zoning designs, like our natural neighbourhoods, becomes a way to resolve the consequences of geographic and methodological scale, the former describing the geography used to identify social processes and the latter the scale of data collection and aggregation [63].

The use of three different spatial representations confirms no measurable effect of NO_2 _exposure on respiratory health in Ottawa. Under all three spatial structures, bivariate OLS regression between the *Mean NO_2 _concentration *and the *Respiratory health outcome rate *suggests that no significant relation is present. Based on the results obtained, we can confirm that in Ottawa, the lack of a significant statistical association is probably not induced by the use of a particular geography. The use of sensitivity analysis allows us to validate and conclude that the strength of the measured relationship is not produced by neighbourhood boundaries poorly reflecting "the ecological properties that shape" health processes [64].

Our methodological approach demonstrates that many factors could explain the observed differences in respiratory morbidity rates in Ottawa. Considering that respiratory health has already been associated with SES in several studies [65], our research serves as another example of the importance of the social and the environmental context on health.

There have been few studies on the role of spatial representation in air quality and health. To our knowledge, this is the first study specifically interested in the effect of scale and zoning on the relationship between exposure to NO_2 _and health. However, our observations do agree with previous research on the subject. Of the limited research on the issue of scale and zoning, others have concluded that "the use of different specifications to assess spatial concentration, agglomerations economies, and trade determinants produces substantial variation in the estimated coefficients" [25]. A study on the relation between morbidity and deprivation showed that the use of spatial representations other than the census tract produced different analytical results [27]. On the other hand, some suggest that the method of neighbourhood definition does not significantly alter relationships and their strength [2,11]. Additional studies on the impact of using different conceptualizations of the neighbourhood on analytical results are required to understand the role of spatial representation. In return, a thorough understanding of the role of MAUP on the study of the relationship between NO_2 _concentration and health will allow decision-makers to develop interventions where they are the most needed. Policy makers' decision about how to improve the health of communities should be strongly influenced by the conclusions that neighbourhood quality affects health. This is particularly of interest in Canada where the population at large believes that the government has a responsibility for the health of citizens [66].

The use of an optimal zone design that has been reviewed and approved by city planners, public health practitioners, community health and resource persons as well as representatives from grassroots organizations is a strength of this research. These natural neighbourhood units have also been used for the Ottawa Neighbourhood Study (http://www.neighbourhoodstudy.ca) and have been updated since their production to reflect changes in Ottawa's communities. Another strong point of this research is the use of an automated zone design to create an aggregated structure that we could use to compare to the census tract structure and our natural neighbourhoods. However, our aggregated structure had to be created by grouping census tracts because of the availability of health data at that geographic level. As a consequence, we did not have as much flexibility when creating the aggregated structure as would be available if a smaller geographic set was used as the basic spatial unit for reporting census and health data. Moreover, our aggregation method produced a single boundary realization that is one of a finite number of possibilities when using a random seeding method that begins with a fixed tessellation defined by census tracts. Future work could provide tools to exhaust all possible aggregations and generate empirical frequency distributions of statistical estimates that could be used to evaluate the sensitivity of results to aggregation effects. Another feature that makes this study more complicated to administer is the fact the NO_2 _concentration is not a statistically significant explanatory variable in the multivariate regressions. If the circumstances were different, we would have a better insight into how NO_2_'s association with respiratory health varies at different scales. Finally, as a cross-sectional type study, we were limited in having only a few weeks of atmospheric sampling and our results do not preclude the future research with more environmental data through time from coming to different conclusions on the NO_2_/Health connection on Ottawa.

## Conclusions

The natural neighbourhoods used in this research can be viewed as "exposure areas" as they were delineated with the objective of creating homogeneous units from an SES perspective [67]. The use of area level data such as income, education and housing variables from the Canadian Census created units where environmental and social conditions are equivalent. There is increasing evidence that neighbourhood context affects the health of individuals living therein [68] and it is not unreasonable to assume that an appropriate delineation of neighbourhoods is essential to research outcomes and recommendations that may arise from such studies. Area units should be delineated with the purpose of representing the expected relationship between neighbourhood and health. If this relationship is well defined, the modifiable area units are not a problem [31]. This research confirms the conclusions of previous studies that more research on the role of spatial representation in health studies in general.

## List of abbreviations

Abbreviations include in the manuscript include the AIC: Akaike Information Criterion; DA: Dissemination area; AD: Discharge Abstract Database; ESDA: Exploratory spatial data analysis; LUR: Land-use regression; MAUP: Modifiable Area Unit Problem; MOHLTC: Ministry of Health and Long-Term Care; NACRS: National Ambulatory Care Reporting System; NO_2 _- Nitrogen dioxide; OLS: Ordinary Least Squares; ONS: Ottawa Neighbourhood Study; SES: Socioeconomic status

## Competing interests

The authors declare that they have no competing interests.

## Authors' contributions

MPP performed the statistical analysis and drafted the manuscript. MCS programmed the automated zone design system and critically revised the manuscript for important intellectual content. All authors contributed equally to the study design, read and approved the final manuscript. This research is a contribution to the Ottawa Neighbourhood Study (http://www.neighbourhoodstudy.ca) and the authors thank all those involved in its inception and continuance, including funders.
